# Invasion and Persistence of Infectious Agents in Fragmented Host Populations

**DOI:** 10.1371/journal.pone.0024006

**Published:** 2011-09-30

**Authors:** Marieke Jesse, Rupert Mazzucco, Ulf Dieckmann, Hans Heesterbeek, Johan A. J. Metz

**Affiliations:** 1 Faculty of Veterinary Medicine, Utrecht University, Utrecht, the Netherlands; 2 Evolution and Ecology Program, International Institute for Applied Systems Analysis, Laxenburg, Austria; 3 Institute of Biology and Mathematical Institute, Leiden, the Netherlands; University of Utah, United States of America

## Abstract

One of the important questions in understanding infectious diseases and their prevention and control is how infectious agents can invade and become endemic in a host population. A ubiquitous feature of natural populations is that they are spatially fragmented, resulting in relatively homogeneous local populations inhabiting patches connected by the migration of hosts. Such fragmented population structures are studied extensively with metapopulation models. Being able to define and calculate an indicator for the success of invasion and persistence of an infectious agent is essential for obtaining general qualitative insights into infection dynamics, for the comparison of prevention and control scenarios, and for quantitative insights into specific systems. For homogeneous populations, the basic reproduction ratio 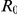 plays this role. For metapopulations, defining such an ‘invasion indicator’ is not straightforward. Some indicators have been defined for specific situations, e.g., the household reproduction number 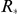. However, these existing indicators often fail to account for host demography and especially host migration. Here we show how to calculate a more broadly applicable indicator 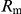 for the invasion and persistence of infectious agents in a host metapopulation of equally connected patches, for a wide range of possible epidemiological models. A strong feature of our method is that it explicitly accounts for host demography and host migration. Using a simple compartmental system as an example, we illustrate how 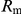 can be calculated and expressed in terms of the key determinants of epidemiological dynamics.

## Introduction

For the prevention, control, and potential (local) eradication of infectious disease agents, it is important to quantify the ability of the infectious agent to invade into a naïve host population, as well as its ability to persist in such a population. On this basis, one can then compare the effects of different scenarios. In homogeneous populations, the so-called basic reproduction ratio 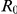 is widely used for this purpose, making it arguably the most important quantity in the study of the dynamics of infectious diseases. It is defined as the expected number of secondary cases caused by one infected host in an otherwise uninfected host population [1]. This ratio also provides an endemicity threshold: if 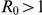, each infected host infects on average more than one other host and, as a result, it becomes likely that the infectious disease will spread in the population (in most models, this will imply persistence of the disease). Conversely, if 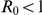, the infectious disease will fade out. A framework for defining and computing 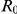, based on how infected individuals spread from one generation to the next, was introduced by Diekmann et al. [2], and has since then been referred to in the epidemic-dynamics literature as the ‘next-generation approach’ [3].

A ubiquitous feature of natural host populations is, however, that they are not homogeneous. Often, they have a fragmented spatial structure in which relatively homogeneous local populations are connected by the relatively rare migration of hosts. Such populations are formed, for example, by humans living in cities [4], cattle living in herds on farms [5], [6], or wildlife populations, such as water voles in the U.K. [7], the Iberian lynx [8], or great gerbils in Kazakhstan [9]. Also populations of plants [10] and fungi [11] are typically structured in space. These spatially structured populations are being extensively studied by means of metapopulation models.

Metapopulation models assume that a population is spread out over a network of patches, each without significant internal structure, and that these patches are connected to each other by the inter-patch dispersal of individuals [12], [13]. Such models are studied both in ecology and in epidemiology. In ecology, typical questions are whether a species can establish a viable population [14], or is able to compete successfully with an already established one [13]. In epidemiology, one of the points of interest is the invasion of an infectious agent into a fully susceptible host population, and the possibility for its subsequent persistence [15], [16], [17].

In a fragmented host population, the basic reproduction ratio 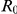 is not a suitable measure for assessing the potential for the invasion and persistence of an infection. This is because, even if the infection would be likely to die out in each patch if patches were unconnected, it can still persist in the metapopulation if the infection spreads among patches faster than it dies out locally. Conversely, although 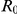 can characterize invasion success within a patch, it cannot predict whether an infectious agent can invade a metapopulation as a whole. For this, the agent needs to be sufficiently efficient in infecting other patches. While the processes involved in this play no role in the characterization of 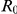, they are key to understand the spread of infections in metapopulations.

For wildlife infections, habitat fragmentation has been shown to be important for determining infection dynamics; this applies, in particular, to processes involved in the spread of zoonotic infections from wildlife to humans [18], [19], [20], and to the evolution of infectious agents. Assessing the impacts of habitat fragmentation, obtaining insights into the underlying epidemiological mechanisms, and, even if only qualitatively, evaluating alternative options for intervention and control, all require a suitable quantitative indicator of an infection's potential for invasion and persistence. As a concrete and practically important example, one can think of attempts to vaccinate a fragmented wildlife population so as to prevent the spill-over of an infection to either humans or domestic animals (e.g., in the case of badgers or possums as sources of bovine tuberculosis). In such situations, 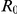 will not be a good indicator of the required vaccination effort. This is because, in addition to the local disease dynamics, the host's connectivity structure and associated dispersal dynamics are crucial determinants of an infection's spread [13]. Broadly speaking, factors affecting invasion either relate to within-patch dynamics (such as contacts between individuals, transmission routes and rates, life-history states, individual heterogeneity, and infectious period) or to between-patch dynamics (such as the connectedness of patches and factors changing the migration of hosts). Which of these factors are particularly relevant for an infection's spread will depend on the particularities of the considered biological system. Whatever the specific factors involved, what is needed is an indicator that can take all relevant factors into account, and thus assume, for spatially fragmented settings, the important role that 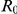 plays for analyzing infection dynamics in homogeneous host populations.

For populations inhabiting a finite number of patches, with each patch being occupied by an infinitely large number of individuals, Fulford et al. [21] investigated the basic reproduction ratio based on the next-generation approach, using a matrix representation. The elements of this matrix represent the expected number of new infected hosts of one type caused by a single infected host of another type. This matrix thus accounts for heterogeneity among individuals, based on considering hosts to be of different types when they occupy different patches. The dominant eigenvalue of this matrix then characterizes invasion success. The approach of Fulford et al. [21] extended work by Hess [22], who assessed the influence of specific spatial arrangements of a small number of patches, while also assuming infinitely many individuals per patch. However, assuming infinitely large populations in each patch is often not appropriate – for example, for a wildlife population structured in small (family) groups or in the case of humans living in households – even though this assumption becomes increasingly suitable as the considered groups of individuals are getting sufficiently large.

For populations structured by (possibly small) group size, so-called household models, Ball [23] introduced an indicator 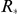 based on an idea by Ball et al. [24]. These models consider a large number of households of constant size, and two types of contacts among individuals: local (or within-household) contacts and global (or between-household) contacts. The measure 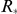 is the household-level analogue of 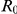 [25], defined as the expected number of households infected by one infected household in an otherwise susceptible population. In an analogous manner, other reproduction numbers have been defined, e.g., to account for overlapping groups, such as workplaces and schools [26], to allow for various household sizes [27], to describe households exchanging infections on a clustered contact network [28]. The same framework has also been adapted to study the effects of different control strategies, such as vaccination [29]. Furthermore, 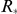 has been applied to study the spread of influenza [30] and measles [31], and a numerical method has been developed for its efficient calculation for infections with waning immunity [32]. However, migration of hosts is an essential ingredient of many wildlife systems and the current household reproduction numbers, constructed with human populations in mind, cannot account for this.

An approach to this problem that works for finite local populations and allows accounting for migration between such populations can be found in evolutionary biology. Specifically, Metz and Gyllenberg [33] investigated how to predict the success of a mutant phenotype invading a metapopulation of residents phenotypes structured into a large number of patches inhabited by finite numbers of individuals, while explicitly accounting for the migration of individuals between patches. This is achieved by defining an invasion indicator 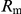 as the expected number of secondary mutant immigrants produced by a patch that has been invaded by a single mutant. This invasion indicator has a threshold at 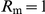: for 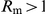, there is a possibility that the number of mutants in the metapopulation increases, so that mutants can invade the population, whereas for 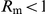, the mutants are expected to die out. The calculation of this invasion indicator is conceptually closely related to an 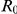-calculation based on a next-generation matrix. The purpose of the present study is to adapt this invasion indicator 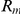 for epidemiological models by replacing the distinction between residents and mutants made by Metz and Gyllenberg [33] with a multi-compartment population structure that can represent life-history- and infection-related states and changes between them, including infection and recovery.

Although the conceptual framework devised by Metz and Gyllenberg [33] is general, they only give calculation recipes for the case of unstructured within-patch populations. The case of infinitely large local populations was further examined by Gyllenberg and Metz [34]. Parvinen and Metz [35] describe an extension to the invasion of mutants in diploid populations, with two types of mutant dispersers, heterozygotes and homozygotes. Massol et al. [36] reinterpret the invasion indicator of Metz and Gyllenberg [33] as a population dynamical threshold parameter, and provide a mathematically rigorous presentation, which, on an abstract level, also covers discrete population structures and multiple disperser types. An equally rigorous (but possibly less accessible) version of the latter result can already be found in Chesson [37].

In an epidemiological context, 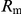 can – in addition to its main use in comparing control options for a specific infection in a specific metapopulation – be used, for example, to study the invasion of a mutant infectious agent into a population in which other infectious agents are already present. The former agent might have a slightly different effect on the host species, resulting, e.g., from a different transmission rate. The indicator 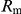 then helps determine whether or not this mutant infectious agent can invade and spread in the resident population, thus enabling studies of the adaptive evolution of infectious agents.

The present paper shows how to calculate 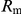 for a general compartmental system of the kind naturally arising in epidemiological dynamics. To aid readers interested in applying our general approach to specific systems, we illustrate our results by studying the simplest disease-metapopulation compartmental system, consisting of only two compartments, one for susceptible and one for infected hosts, with infected hosts becoming susceptible again after recovery. To maximally bring out the effects of habitat fragmentation, we purposely develop this example in a setting that remains as close as possible to the idealization of an infinite homogeneously mixing (mass-action) population that underlies the definition and calculation of 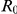, but with the crucial difference that the population we study is fragmented into finite populations inhabiting an infinite number of habitat patches that are equally connected through inter-patch dispersal.

## Methods

### Infection invasion in a general compartmental system

Our objective is to study the invasion of an infectious agent into a fragmented population of susceptible hosts that has an implicit spatial structure: we assume that the host population inhabits an infinite number of identical patches, with each patch being equally connected to all other patches and containing a finite number of hosts. We expect such a structure to provide a reasonable idealized model for infection scenarios in which the number of patches is large and long-range host dispersal is frequent enough for an infection to move from any given part of the landscape to any other in relatively few steps. To highlight the features of suitable systems, we can think of the great gerbils in Kazakhstan [9], living in family groups in underground burrow systems, in which the plague bacterium *Yersinia pestis* spreads. In the resultant gerbil metapopulation, short-range host migration occurs for establishing new family groups and for foraging. In addition, there is long-range dispersal, by birds, of the pathogen across the entire landscape of burrow systems.

We build on general ideas for studying invasion fitness in compartmental systems, introduced already in [1], [2] in a much broader setting, and more recently reviewed for compartmental systems by Diekmann et al. [3]. Those earlier expositions already accounted for heterogeneity among host individuals by allowing for an arbitrary number of ‘types’ of hosts in terms of host features that can be relevant for the infection dynamics, such as ageclass, development stage, or sex. On that basis, a next-generation matrix was defined, the elements of which give the expected number of new infected hosts of one type caused by a single infected host of another type. This matrix does exactly what its name suggests: it gives the next generation of infected individuals, distributed over all possible infected host types, starting from that distribution in the current generation. The basic reproduction ratio 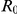 is then obtained as the dominant eigenvalue of this matrix, and functions as an indicator for the growth or decline of the total number of subsequent generations of infected hosts upon iterating the matrix. Here we integrate this general approach to compartmental models with the framework for defining invasion fitness in spatially implicit metapopulation dynamics introduced by Metz and Gyllenberg [33].

#### Metapopulation dynamics

A compartmental model in epidemiology classifies each individual into one compartment, or state, at any given point in time, where the various compartments correspond to the different stages in the course of the infection within an individual, and possibly in the individual's life history. So, for example, one may consider a susceptible juvenile female, or an infectious adult male. In compartmental models, the switch between individual states is instantaneous. If a more gradual change is called for, e.g., for describing changes in the severity of the disease, additional consecutive compartments can be introduced, or a continuous description based on integral equations can be employed (for epidemiological examples, see [1]). The assumed transition rates between states specify a system of dynamical equations. Births into at least one compartment, and deaths in all compartments, are typically also considered. Here, we augment these within-patch dynamics with equations that describe emigration from and immigration into patches. The terms “demographic dynamics”, “infection dynamics”, and “migratory dynamics” can be used to distinguish the parts that describe, respectively, the transitions between “normal” or infection-free life-history states, including birth and death rates, the transitions that involve states associated with the infection, and the migration events. An individual's compartment fully characterizes its (dynamically relevant) state. Below we consider models with 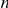 compartments in a convenient ordering: the first 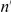 compartments describe the infection-free life states of an individual and the remaining 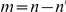 compartments describe its infection-related states. In the case of the invasion of infection in populations of great gerbils in Kazakhstan, we could consider three compartments (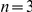): one for susceptible, one for infectious, and one for recovered gerbils (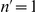 and 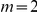).

The state of a patch can be described by a vector 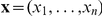 specifying the numbers of individuals inhabiting this patch that belong to each of the 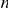 considered compartments. Since local populations within patches have a finite size, with a maximum occupation of 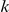 individuals, there is only a finite number 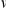 of possible patch states, and counting these states gives
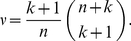
(1)For any application, one can define a bijective map from the set of possible indices 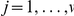 to the set of possible patch states 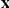. Accordingly, we can speak either of a patch state 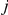 or of a patch state 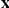, and we will use either as a subscript depending on what seems more informative. For convenience, the ordering of patch states is again such that the first 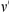 patch states are infection-free, and the remaining 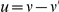 patch states are infection-related.

Apart from their differential occupation by individuals, all patches are assumed to be equivalent. The state of the metapopulation can therefore be described by specifying, for each possible patch state 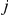, the fraction 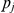 of patches currently found in this state. The resultant 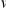-vector 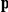 thus has non-negative components that sum to 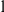. In addition, we introduce a disperser pool to keep track of the individuals in each of the 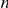 compartments that have emigrated from one patch and have not yet immigrated into another. The state of the disperser pool is described by an 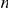-vector 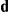, which we keep normalized so that its components 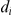 can be interpreted as the average number of dispersers per patch in compartment 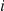. We thus employ a general and very flexible way of modeling dispersal, through which the biology of any specific system can be respected by specifying how long individuals on average stay in the disperser pool and what can happen to them during that time. Effectively, this just involves a simple bookkeeping of individuals while they are not residing in a patch. In the case of great gerbils, one should think of the disperser pool being populated by individuals searching for a suitable empty patch to establish a new family.

The full metapopulation dynamics can be compactly described by the following system of ordinary differential equations,
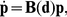
(2a)

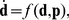
(2b)where the dots denote time derivatives and with 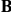 and 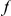 as specified below. The 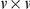-matrix 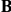 contains the transition rates. These depend on the state of the disperser pool, because this determines the number of individuals currently available for immigration. An off-diagonal element 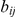 of 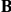 measures the rate at which a patch in state 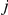 transforms into a patch in state 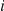. A diagonal element 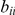 of 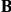 measures the total transition rate at which a patch in state 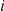 transforms into any other state 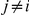. It is therefore negative, as it describes a flow out of patches in state 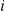, and consequently, the columns of 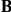 add up to 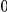. The 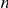-vector-valued function 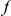 describes the dynamics in the disperser pool and is assumed to have the general form
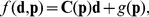
(2c)in which an 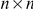-matrix 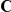 describes mortality and transformation in the disperser pool, as well as immigration into patches, the latter possibly depending on patch states, and the function 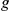 describes emigration from patches. This form leaves room for, e.g., an individual in a latent stage to become infectious before it enters a new patch, or a sick individual to recover; in many specific systems, the length of stay in the disperser pool will be too short for these changes of state to be practically important. We preclude, however, infections from occurring in the disperser pool. This is because in natural systems contacts between hosts in the disperser pool are typically so infrequent that they do not make a significant contribution to transmission. Otherwise, one would have to consider the possibility of the infection becoming endemic in the disperser pool, which is beyond the scope of the 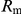-calculations developed here.

Based on this framework, we now investigate the fundamental question whether an infectious agent can spread in an established host metapopulation and become endemic. For terminological convenience, we will distinguish between 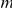 “invader types”, “invader compartments”, or “invaders”, and 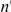 “resident types”, “resident compartments”, or “residents”.

#### Reinvasion cycle

The possibility for long-term persistence of certain invader types in an existing environment can be inferred from their full population dynamics, i.e., from their transition rates between compartments and patch states. If there is but a single attractor of the resultant dynamics, we can infer their long-term persistence in a simpler manner, by investigating the population dynamics of the invaders while they are rare. This simplification is possible because, whatever happens when the invaders become more abundant, they would again have to become rare before going extinct.

**Figure 1:**
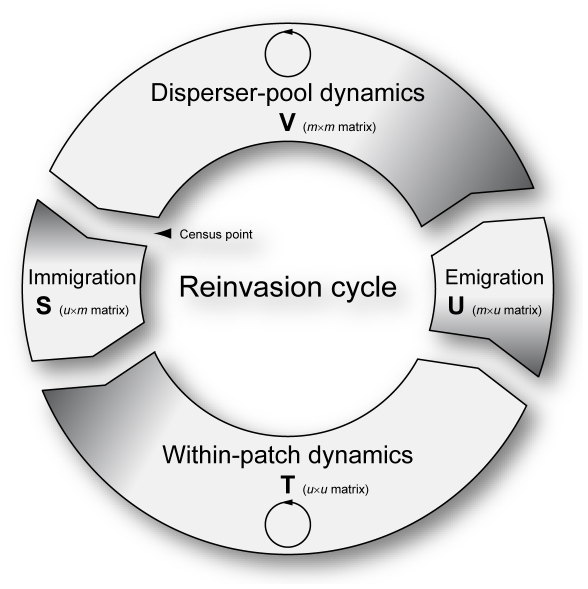
Reinvasion cycle. The dynamics of invaders in a metapopulation depend on within-patch and disperser-pool dynamics, coupled through dispersal behavior. The cycle of immigration of invaders into a set of patches, production of new invaders within those patches, emigration of invaders into the disperser pool, survival and transformation of invaders within the disperser pool, and, finally, re-immigration of invaders into the patches, can be broken up into four stages as shown. The processes taking place in the four stages are described by the matrices 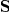, 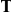, 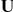, and 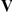. These are multiplied to yield the matrix 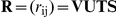, whose elements describe the expected number of secondary invasions by invaders of type 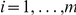 resulting from a primary invasion of invaders of type 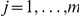. Generalizing the key role 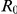 plays for the analysis of infectious diseases in unstructured host populations, the dominant eigenvalue 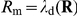 measures the factor by which the total number of invaders grows during each turn of the cycle. Thus, 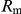 exceeds 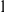 if and only if the invader population expands, making it a natural invasion indicator. The construction and interpretation of the four matrices is explained in the text.

Whether invaders become more or less abundant depends both on their dynamics within patches and on their emigration, dispersal, and immigration into new patches. This “reinvasion cycle” (Fig. 1) is at the focus of all our analyses below.

The assumption of invader rarity considerably simplifies the population dynamics of invaders. As long as invaders are rare in the metapopulation, it is very unlikely that a once-invaded patch will be invaded again. Consequently, within-patch dynamics can be examined while being temporarily undisturbed by the arrival of more invaders from the disperser pool. Also the effect of rare invaders on the distribution of patch states is negligible; therefore, the population dynamics of the dispersers is approximately linear.

To define invader dynamics efficiently, it is helpful to monitor, or census, the invaders at the “narrowest” point of the reinvasion cycle. Since the within-patch dynamics (Eq. 2a) has many more states than the disperser-pool dynamics (Eq. 2b), we census the invaders as they leave the disperser pool.

For the models considered here, the reinvasion cycle can be decomposed into four stages (Fig. 1), each of which is described by a matrix:

- Immigration of invaders from the disperser pool and distribution over patches (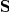).
- Population dynamics within invaded patches (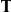).
- Emigration of invaders from patches and collection into the respective compartments of the disperser pool (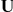).
- Population dynamics of invaders in the disperser pool (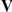).

This sequence of stages thus describes how an invader leaving the disperser pool contributes to future invaders leaving the disperser pool. The latter individuals may include the former individual or comprise just its offspring. Therefore, the reinvasion cycle need not correspond to the life cycle of an invader individual, which, in general, might go through the reinvasion cycle partly or repeatedly. Furthermore, while transitions between the four stages are fully stochastic, the four matrices above quantify the resultant deterministic expectations: 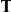 and 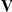 describe the expected total sojourn times (i.e., durations of stay) in the two dynamical stages, whereas 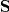 and 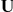 describe the expected rates of transition through the two migration stages.

The product
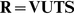
(3)is a dimensionless 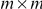-matrix, with 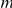 being the number of invader compartments (an efficient procedure for obtaining the matrix product 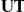 is given in Appendix S2). The elements of 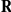 describe the expected number of secondary invasions of invaders in a given compartment resulting from the primary invasion of a single invader in a (potentially different) compartment. Following Metz and Gyllenberg [33], we obtain the factor by which the invader population is expected to grow during one reinvasion cycle as the dominant eigenvalue of this matrix,
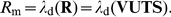
(4)If 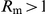, the invader can invade, whereas if 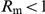 it cannot. Throughout the remainder of this study, we therefore refer to 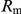 as the invasion indicator.

Another natural decomposition of 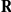 would be
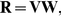
(5a)with 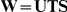 describing the processes involving patches and 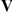 describing the processes taking place solely in the disperser pool. Both 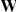 and 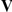 are 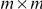-matrices; the fact that one can reverse their order of multiplication without changing the dominant eigenvalue, 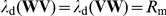, mathematically reflects the biological fact that the invasion indicator is unaffected by the choice of census point. Analogously, if we chose our census point after immigration into, or before emigration from patches, we would end up with a product of two 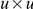-matrices, with 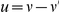 being the number of invader patch states, which again possesses the same dominant eigenvalue 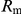. In summary, all four possible census points yield the same result.

Instead of the decomposition into dimensional matrices 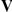 (with elements having the unit of time) and 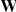 (with elements having the unit of rate, or time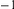) discussed above, one can alternatively consider a similar decomposition,
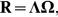
(5b)into dimensionless matrices 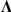 and 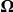. These matrices have a direct individual-based interpretation: for all pairs of invader patch types, the elements of 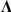 are the expected numbers of immigrants into a patch per emigrant from a patch, and the elements of 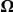 are the expected numbers of emigrants from a patch per immigrant into a patch. Since such an individual-based perspective is preferable in some studies, below we will mention also how to construct 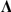 and 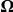.

#### Invasion indicator

Given a metapopulation state 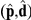 that describes a resident population at its interior equilibrium, we consider the arrival of an invader in an arbitrary patch. When the maximum patch occupancy is finite, eventual extinction of the invader type within any one patch is certain. Before this happens, however, invader types may migrate from a patch into the disperser pool, and eventually arrive in new patches. We now quantitatively analyze the resultant reinvasion cycle (Fig. 1).

We recall that the resident is described by the 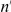 infection-free compartments and 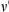 infection-free patch states, while the invader is described by the remaining 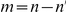 infection-related compartments and 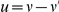 infection-related patch states. We can thus refer to the 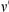 patch states as invader-free states and to the 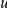 patch states as invader states.

#### Immigration and distribution over patches

The 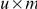-matrix 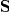 describes immigration from the disperser pool into patches. Its elements 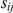 are the expected rates at which an invader of type 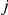 in the disperser pool creates a patch in invader state 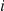. To facilitate the calculation of 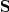, we decompose this matrix as 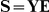, so that the diagonal 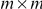-matrix 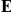 specifies the rates 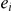 at which an invader of type 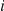 encounters patches, and the 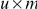-matrix 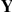 specifies the probabilities 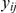 that the arrival of an invader of type 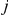 in a random patch creates a patch in invader state 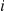.

Since invader types are assumed to be rare, invasions creating a patch state with more than one invader can be neglected, so the distribution of patch states as it presents itself to the invader encountering patches at random is approximately given by 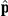. To determine 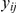, we introduce the probabilities 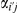 that an invader of type 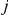 enters a patch in invader-free state 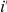. Therefore, if 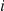 is an invader patch state with exactly one invader of type 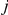, we obtain
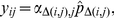
(6)whereas 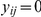 otherwise. Here the function 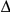 turns an invader patch state 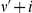 with exactly one invader of type 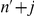 into the corresponding invader-free patch state 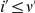 by removing that invader. The immigration rates of the 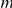 invader types are given by the 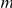 row sums of 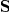; these depend on 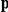 and can be assembled in a diagonal 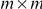-matrix 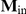 with 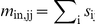.

#### Within-patch dynamics

The 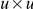-matrix 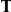 describes the outcome of within-patch dynamics. Its elements 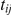 are the expected total sojourn times (i.e., durations of stay) in invader patch state 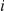, given an initial invader patch state 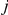. 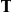 is obtained by integrating over a matrix 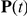 of time-dependent probabilities 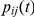 to find a patch in state 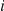 at time 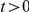 that had initially been in state 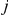 after invasion at 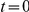,
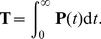
(7)Because each element of 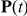 is smaller than 1, the matrix is exponentially bounded, and the expected total sojourn times are thus finite.


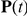
 is obtained by solving the part of Eq. 2a corresponding to the invader patch states while the resident patch states are fixed at the equilibrium 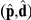 of the invader-free resident population. This implies that in Eq. 2a the resident immigration rates are constant in time, determined by 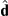, and the invader immigration rates are 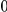, reflecting that secondary immigration by invaders can be neglected due to their initial scarcity. Denoting the 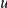-vector of invader patch frequencies as 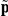 and the corresponding 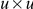-submatrix of 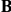 as 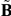, we thus have
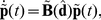
(8)with the straightforward solution
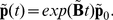
(9)The initial states 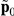 of interest to us are those in which the patch is with certainty in a given invader state, i.e., 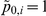 for a given state 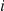 and 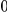 otherwise. Jointly, all these 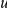 initial states, when arranged as column vectors in a matrix and properly sorted, are thus represented by the 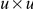 identity matrix. Therefore,
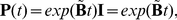
(10)which yields
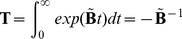
(11)for the matrix of expected total sojourn times.

#### Emigration and collection into compartments

The 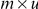-matrix 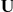 describes emigration from the patches into the various compartments of the disperser pool. Its construction is similar to that of 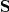, except that the disperser pool is “encountered” with certainty and that emigration rates 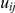 could depend on the patch state 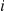.

Often, however, the simplifying assumption can be made that emigration rates depend only on the emigrant's type. In this case, we decompose 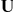 as 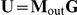, so that the diagonal 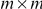-matrix 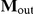 specifies, for each invader type, the emigration rate into the disperser pool, and the 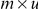-matrix 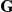 describes the associated collection into invader compartments. The elements 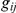 are the number of invaders of type 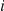 in invader patch state 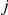. This matrix is easily constructed from the correspondence of patch-state indices and patch-state vectors, by arranging as column vectors in the appropriate 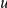 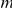-subvectors 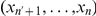 of the 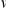 possible patch-state vectors 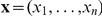.

#### Disperser-pool dynamics

Finally, we consider the 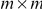-matrix 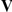, whose elements 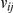 describe, for invaders arriving in invader compartment 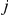 of the disperser pool, the expected time spent in invader compartment 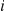. This matrix depends on the function 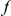 in Eq. 2b, which in most practical cases will be of the form in Eq. 2c. Reduced to invader states, this yields
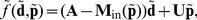
(12)where the 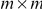-matrix 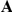 describes the state transitions and death rates of individuals in the disperser pool. The expected total sojourn times in the disperser pool are then given by
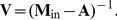
(13)


#### Dimensionless matrices

The dimensionless matrices describing, respectively, for the various invader types the expected number of immigrants into a patch per emigrant from a patch, and the expected number of emigrants from a patch per immigrant into a patch (Eq. 4b), can be constructed from the above matrices as 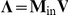 and 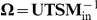. Again, 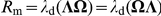.

#### Viability and endemicity

With all these matrices in place, we can determine 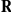 and find its dominant eigenvalue (Eq. 3), to obtain the invasion indicator 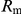 of the invader.

In addition to studying the invasion of an infectious agent into an established host population, we can also study if a host population can viably establish itself in a patch structure in the absence of the infection. As already pointed out by Massol et al. [36], both of these questions concern a specific kind of persistence and can be answered using the same formal procedure of calculating an invasion indicator. In the former case, this indicator describes the invasion potential and viability of the infection-free host, while in the latter case, it describes the invasion potential and endemicity of the infectious agent. To highlight this distinction, we use the symbols 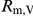 and 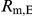 instead of the more generic 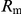, and call the former quantity the “viability indicator” of the host and the latter quantity the “endemicity indicator” of the infectious agent. Despite this distinction, there is a close correspondence in how these quantities are defined:

- To assess viability, we consider the trivial equilibrium, corresponding to an empty metapopulation, and then analyze the invasion potential of an infection-free host. Here the invaders are the infection-free hosts, so we reinterpret 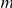 as the number of infection-free compartments while setting 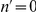

- To assess endemicity, we consider an equilibrium of an infection-free viable host population, and then analyze the invasion potential of an infected host. Here the invaders are the infected hosts, so we interpret 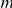 as the number of infection-related compartments and 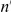 as the number of infection-free compartments.

Accordingly, for assessing viability, we need to consider the trivial equilibrium 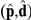 (i.e., 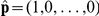 and 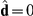), while for assessing endemicity, 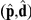 is an equilibrium of an infection-free viable host population, computed from Eqs. 2 in the absence of invaders. Denoting the corresponding within-patch transition matrix and disperser-pool function by 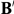 and 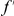, respectively, this yields
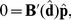
(14a)

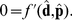
(14b)


Since the coupling of the within-patch dynamics with the disperser-pool dynamics prevents a general solution of this equilibrium, we provide in Appendix S1 an iterative numerical scheme inspired by Metz and Gyllenberg [33]. Before attempting to calculate this equilibrium, it will be good practice first to ascertain the host population's viability by calculating its viability indicator.

#### Summary of procedure

Based on these specifications, we can summarize the suggested procedure for studying the invasion of an infectious agent in to a fully connected metapopulation with explicit host migration:

1. Write down the dynamical equations for the host in the absence of the infectious agent.
2. Calculate the viability indicator 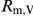 of the infection-free host metapopulation using Eq. 3.
3. For 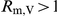, find the non-trivial equilibrium 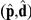 of the infection-free host metapopulation using Eqs. 13.
4. Enhance the aforementioned dynamical equations by incorporating compartments and interactions required to describe the infection of hosts.
5. For 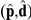 from step 3, calculate the endemicity indicator 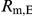 of the infectious agent using Eq. 3.

To illustrate the application of our framework with an example, we now show how to calculate and analyze the invasion indicator 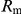 for a simple concrete compartmental system.

### A concrete example: application to a compartmental system

Applying the framework introduced in the previous section, we now study as an example an infectious-disease dynamics described by a simple model with only two compartments, corresponding to a “susceptible” and an “infected” state, respectively, with hosts becoming susceptible again after recovery from infection (a so-called 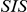-model). Following the steps outlined in the preceding subsection, we show explicitly how to calculate the invasion indicator and express it in terms of the ingredients of the model.

#### Host dynamics

Each patch has a carrying capacity 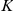 and a maximum occupancy 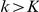. The patch state is described by a single compartment for the number of susceptible hosts; according to Eq. 1, there are, therefore, 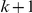 possible patch states. Consequently, the state of the metapopulation is described by a 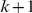-vector 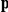 with components 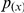 that specify the fractions of patches containing exactly 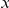 individuals, along with a scalar 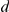 that specifies the number of individuals per patch in the disperser pool. As discussed before, we can use the patch-state vector 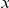 as the subscript of 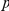; to avoid any mix-ups with subscripts based on the consecutive numbering of patch states, we enclose the 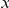 in parentheses.

Patch states change through births, deaths, and migrations. The birth rate 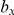 in a patch is logistically density dependent, 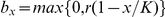, with 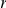 denoting the intrinsic birth rate. We denote the per capita death rate by 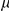, the per capita emigration rate to the disperser pool by 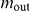, and the patch-encounter rate in the disperser pool by 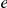. Hosts always enter a patch, unless the patch is already filled to capacity. The metapopulation dynamics (Eqs. 2) of the host is thus given by
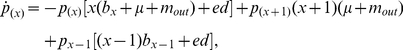
(15a)

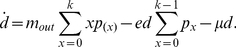
(15b)Eq. 14a formally assumes 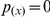 for 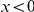 or 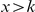, and can also be written in the matrix-vector form of Eq. 2a. As a concrete example for 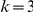, Eqs. 14 become
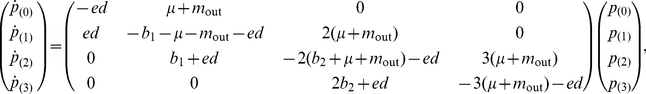
(16a)

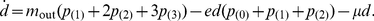
(16b)


#### Host invasion: viability

To determine the conditions under which the host population is viable, we calculate its viability indicator 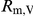, which equals the single element of the 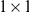-matrix 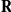 of the infection-free dynamics. Since 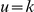 of the 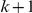 possible patch states contain at least one host individual, the immigration matrix 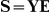 is a 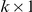-matrix. By the assumption of rarity, a host with certainty invades an empty patch and turns it into a patch containing exactly one host, 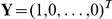. 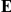 is a 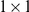-matrix with the patch encounter rate 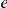 as its single element.

For the calculation of the 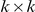-matrix 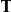, Eqs. 7, together with Eq. 14a, yield
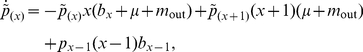
(17)for 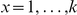, with 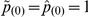, and, again, formally 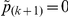. These equations can be rewritten in the matrix-vector form of Eq. 7, whence we can extract the 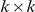-matrix 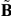 required for the calculation of 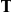 via Eq. 10. For 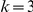, we thus obtain
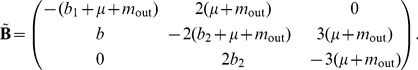
(18)


As for calculation of the emigration matrix 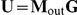, the number of hosts in each invader patch state is given by the 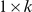-matrix 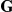 with 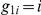, so that 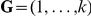. The 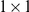-matrix 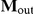 of emigration rates has as its single element the host emigration rate 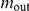.

Finally, the 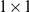-matrix 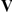 of total sojourn times in the disperser pool has the single element 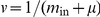, with 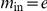 (because all patches are empty, and thus 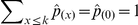), gleaned from the disperser-pool dynamics in Eq. 14b. We can now multiply all these matrices according to Eqs. 3, using Eq. 10, to obtain the viability indicator,
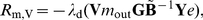
(19)which, in our example with 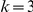, yields
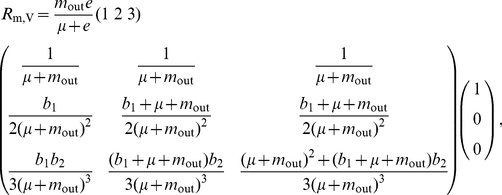
(20a)and thus
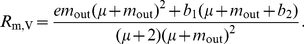
(20b)


**Figure 2:**
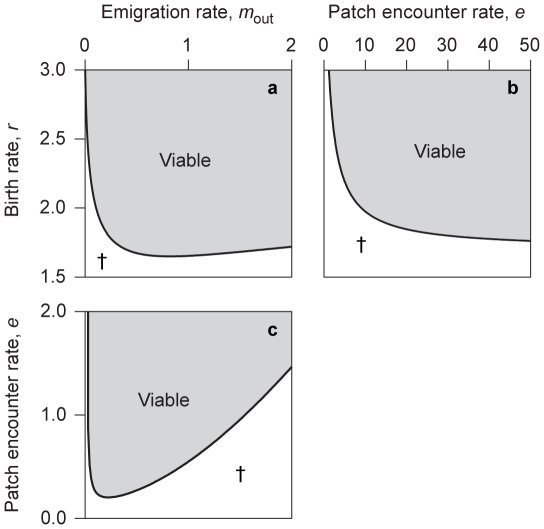
Illustration of an analysis of the viability indicator 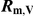. The indicator is shown as a function of (a) emigration rate and intrinsic birth rate, (b) patch encounter rate and intrinsic birth rate, and (c) emigration rate and patch encounter rate. Parameter regions in which the uninfected host population is viable (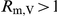) are highlighted by shading, while regions in which it goes extinct are marked by a cross (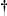). Other parameters: 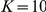, 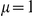, 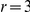, 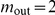, and 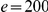.

The explicit expression now available for 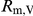 through Eq. 19b, enables the easy numerical analysis of host viability. An example of such a study is illustrated in Fig. 2, where the influence of the intrinsic birth rate, emigration rate, and patch-encounter rate, on the viability indicator is shown by fixing one of these parameters to a default value and varying the other two. For this purpose, time is rescaled so that the death rate equals 1, 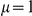, meaning that all parameters involving the unit of time are expressed relative to the lifespan 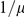 of the host, where 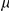 denotes the un-scaled death rate. Furthermore, we arbitrarily set the carrying capacity to 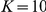, because we are interested in the dynamics of small populations. The default patch-encounter rate is chosen such that individuals do not spend a long time in the disperser pool, and no dynamics occur in this pool except deaths. The default emigration rate is chosen so as to describe hosts migrating on average twice during their lifetime. The default intrinsic birth rate is chosen such that each host gives on average birth to three other hosts.

Only for 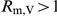, the infection-free host population is viable. For model parameters fulfilling this condition, we determine the interior equilibrium 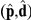 of the infection-free host population as a stationary solution of Eqs. 13, using the numerical scheme described in Appendix S1. On this basis, we can proceed with studying the invasion of the infectious agent.

#### Infection dynamics

In addition to the compartment representing susceptible hosts, we now introduce a second compartment (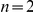) that represents infected, and in our case also infectious, hosts (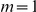). According to Eq. 1, this results in 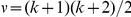 different patch states, of which 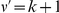 contain no infected hosts, and 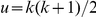 contain at least one infected host. Thus, each patch state can be described by a 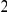-vector, 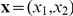, where 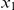 represents the number of susceptible hosts and 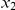 the number of infected hosts. Likewise, the disperser-pool state is now given by a 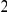-vector, 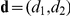.

Hosts are born susceptible, and when they recover from the infectious disease, they are immediately susceptible again. The state of the metapopulation is now described by the fractions 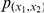 of patches containing exactly 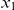 susceptible and 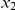 infected hosts. Patches are assumed to be small, so that individuals are saturated in the amount of contacts they have, and the fraction of encounters of a given infected host with a susceptible host thus equals the fraction of susceptible hosts in the patch. We denote the within-patch contact rate by 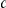, the transmission probability upon contact by 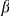, and the recovery rate by 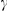. The infection is furthermore assumed to be demographically neutral, in the sense that the migration rates and the mortality of infected individuals are the same as those of susceptible individuals. The corresponding equations for the patch fractions are then
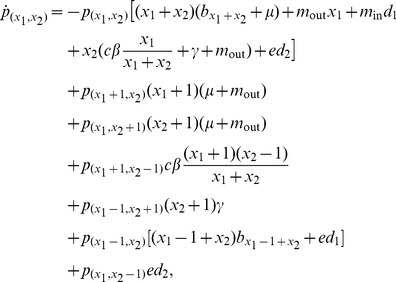
(21a)where we again formally assume 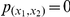 for 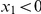, 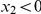, or 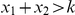. The disperser-pool dynamics are now given by
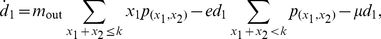
(21b)

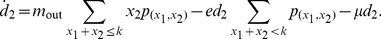
(21c)At the onset of infection invasion, dispersing infected hosts are so diluted by uninfected hosts that contacts among the former can be neglected.

#### Infection invasion: endemicity

Based on the interior equilibrium of the infection-free host population, 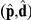, and the metapopulation dynamics specified in Eqs. 20, we can calculate the invasion indicator 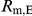.

As before, the matrix 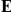 of patch encounter rates has as its single element 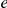. The distribution matrix 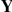 is now a 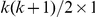-matrix with components 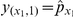 and 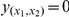 for 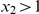 in its single column. For the calculation of the matrix 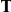 of expected total sojourn times in the patches from Eq. 10, we construct 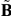 from the reduced Eqs. 20a,
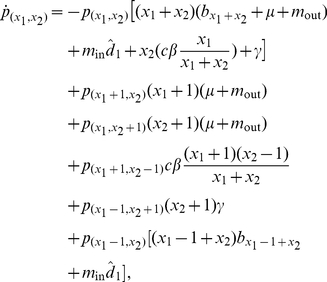
(22)where we again formally assume 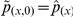 and 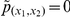 for 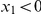 or 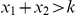 (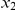 is always positive). The collection matrix 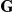 describes the number of infected individuals per infection-related patch state and thus contains a single row with components 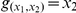. The matrix 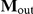 of emigration rates has as its single element 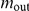. The matrix 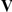 of total sojourn times in the disperser pool likewise again has a single element 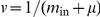, with 
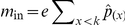
.

**Figure 3:**
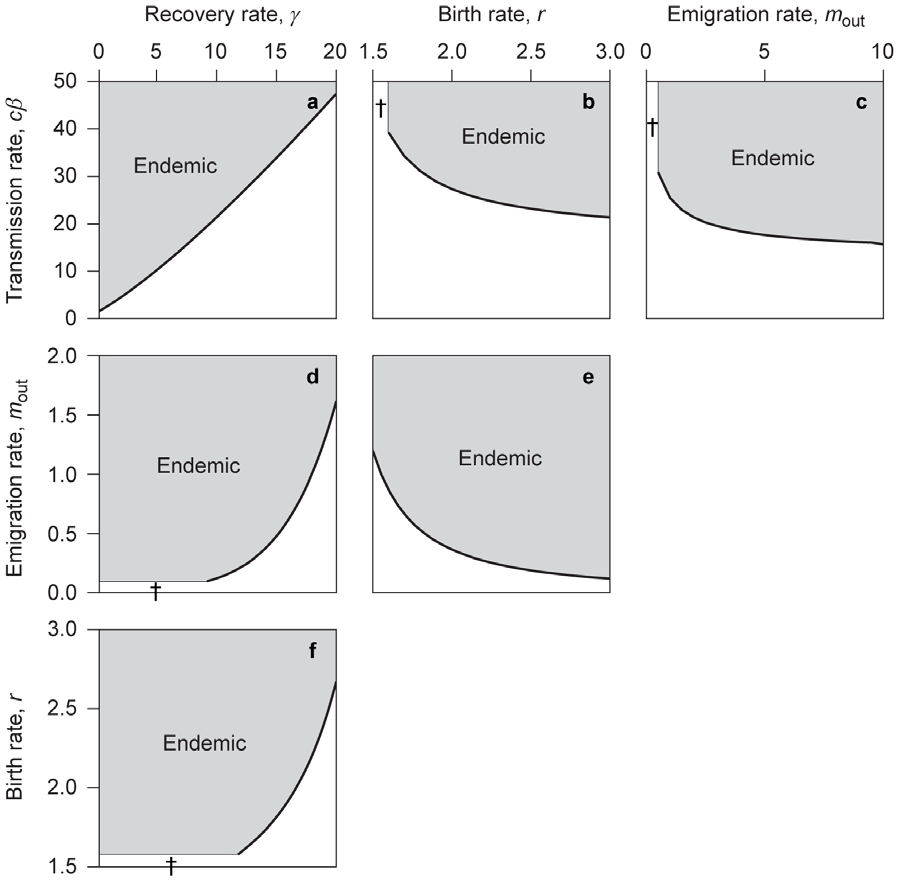
Illustration of an analysis of the endemicity indicator 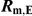. In each panel, the indicator is shown as a function of two parameters. Parameter regions in which the disease can become endemic (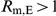) are highlighted by shading. For intrinsic birth rates 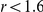 and emigration rates 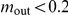, the uninfected host population is not viable (Fig. 2); the corresponding parameter regions are marked by a cross (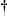). Other parameters: 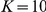, 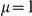, 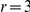, 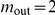, 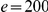, 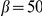, and 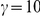.

Finally, although we have no particular biological interest in our example, other than using it as such, we note that one can explore, using these results, the influence of all model parameters (intrinsic birth rate, transmission rate, recovery rate, and emigration rate) on the endemicity indicator 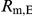 can easily be explored. We illustrate this with the set of two-parameter plots shown in Fig. 3.

## Discussion

In this paper, we have reinterpreted the invasion indicator for a mutant in a metapopulation, introduced by Metz and Gyllenberg [33] and Gyllenberg and Metz [34] in evolutionary biology, as an invasion indicator in infectious-disease dynamics. Explicitly accounting for host migration between the patches of a fragmented host population, we have used this approach to investigate the viability of uninfected host metapopulations, as well as to analyze the invasion and possible endemicity of an infectious agent in such metapopulations. Describing the life-history stages and the infection-related stages of hosts using a general compartment model, our framework is applicable to a very wide range of disease models. As an example, we have demonstrated how to use our framework for studying the invasion and endemicity of a disease in a simple 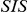-model.

The basic idea of invasion analysis is that to assess the long-term chance of success of a particular type of individual or infectious agent trying to invade a given environment, it suffices to determine its fitness, or basic reproduction ratio, in this environment while the considered type is still rare, and its effect on that environment thus still is negligible. An important assumption underlying this invasion indicator for infectious agents is that the host population is at equilibrium when the infectious agent tries to invade it. This is often a reasonable assumption. But it is also possible that a small host population settles in a new habitat without taking any infectious agents along [38]. Then, when shortly after this host invasion an infectious agent is introduced, the host population is not at equilibrium yet. Instead, we may expect the host population to be overall smaller, with more empty patches and smaller populations in the occupied patches, thus making it less easy for the disease to invade. If such is indeed the case, 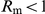 will still preclude invasion, whereas for 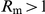 the disease may have to bide some time before its invasion becomes feasible. In this sense, the invasion indicator introduced here is conservative. Under special circumstances, it is possible, however, that invasion cannot occur at equilibrium, 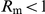, even though it may occur during a phase of host expansion. For example, the infectious agent may target mainly young hosts, of which there may be relatively more during the build-up of a host population. Yet, even in this case 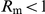 would normally imply that the infectious agent cannot remain endemic once the host population has equilibrated.

In a large class of compartmental systems with susceptible replenishment, 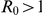 implies persistence, and thus endemicity. Nevertheless, there exist exceptional models in which the infection-free equilibrium is locally stable (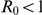), and yet coexists with a stable interior equilibrium does not imply persistence and endemicity in the mathematical sense, but may do so in an intuitive biological sense. Such cases are rare in epidemiology, and appear to occur basically only in models in which behavioral changes play an important role. We therefore expect that for reasonable assumptions about susceptible replenishment (such as the absence of Allee effects), and without infection-related behavioral changes or complicated effects of the immune system, endemicity occurs in metapopulations if and only if 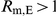 (with endemicity formally defined as the existence of at least one interior attractor). In any case, in practice 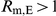 more often than not will serve as a sufficient condition for endemicity. While in principle more severe measures than suggested by the objective 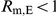 may be necessary to eradicate a disease, the invasion indicator introduced in this study can always be used to assess whether proposed measures have no chance of success.

A metapopulation is characterized by, among other features, the considered patch network and its connectivity structure. Here, we examined the case in which all patches are equally connected to each other. One may question the realism of this assumption, since in reality our implicit spatial structure is replaced by an explicit spatial arrangement of the patches making up the metapopulation, with some patches being farther away from a given patch than others, and thus perhaps less-well visited by individuals emigrating from that given patch. Of course, the assumption of equal connectedness is an idealization, just as the assumption of a well-mixed population underlying many, if not most, models of single populations (also adopted here to describe the mixing of individuals within each patch). In fact, the assumption of a well-mixed population underlies the definition and calculation of 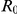, and if we think of patches as individuals that mix, then equal connectedness is the natural translation of having a well-mixed situation at the metapopulation level. The biology of the systems one studies, and the precise questions one studies, will dictate whether such simplifying assumptions are permissible. We agree that metapopulation structures in which each patch is connected to only a handful of neighboring patches may offer a more accurate description of realistic scenarios and lead to qualitatively different behavior than described here. This is because different spatial arrangements tend to result in differences in the spread of an infection, which in turn affects, for example, which control measures are best being taken [21], [22]. Also the rate of spread is influenced by such connectivity [39]. The presence of hub patches in a network, which are highly connected compared to other patches, can also have a profound influence on an infection's spread and persistence, and removing such patches can substantially reduce an infection's basic reproduction ratio [40], [41]. In conclusion, we can interpret the simplifying connectivity assumption made in this study as a limit that will not always be accurate, but offers a natural and generic baseline that is approached quickly when dispersal occurs beyond a patch's immediate spatial neighborhood. In our assessment, the latter applies more often than not.

The assumption of identical patches is made for exposition purposes only. Our approach also works well when a discrete number of different patch types are distinguished. Such types could capture, for example, classes of patch size or patch quality. While one then needs to calculate equilibrium distributions of individuals over the different patch types, the key assumption that the dispersal pool is common among patch types ensures the applicability of our framework [35].

All networks discussed so far comprise habitat patches and connections that remain fixed in time. Yet, habitat structures can change: for example, the degree of fragmentation may increase as a result of human land use involving the building of roads [42], the invasion of a predator [43], or by infestation [44]. Habitat fragmentation and land use have been shown to strongly affect the spread and persistence of infectious diseases. This applies especially to vector-borne diseases and to infections carried by small rodents (e.g., [45], [46]).

In our model, emigrating individuals move through a disperser pool before immigrating into a patch. This pool is not only a bookkeeping device; instead, its introduction allows to study the ecological effects of migration on individuals, such as time lost for reproduction and dispersal mortality resulting from increased vulnerability [47]. While the 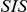-model studied as an example did, for the sake of simplicity, not feature such effects, the framework introduced here readily provides for such additions; the appropriate terms just need to be incorporated into the dynamical equations. Moreover, the time spent in the disperser pool can easily be adjusted, so that it reflects the time a host needs to find a new patch. While our method allows for changes of infection state during an individual's stay in the dispersal pool, for most real systems the average stay will be short compared to the average length of, e.g., the latency or infectious period. Therefore, state changes in the disperser pool may well be negligible in many practical applications.

We also assumed, for convenience, a one-to-one match between the life-history stages occurring in the patches and in the dispersal pool. However, our framework applies in an exactly similar manner when there is no such match, simply by considering the union of those two sets of stages. This occurs, for example, when pathogens move between patches independently of their hosts, as is typical for fungal plant diseases that spread only through seeds and spores. Likewise, in vector-borne infections of animals, it may be the vector that spreads, rather than the host, or vector and host may spread independently. While we have formulated our theory for situations in which host and vector spread together (as in, for example, tick-borne or flea-borne infections), it readily carries over to all such situations. The important point is to specify which types of individuals are involved in the migration between patches and to set up the modeled compartment structure accordingly.

Our framework describes immigration from the disperser pool into the patches through a matrix of expected rates at which individuals of certain types in the disperser pool create patches in certain states. To discuss the construction of such a matrix, we have offered a decomposition of this matrix into a matrix of patch-encounter rates, which we assume to depend only on type and nothing else (although this assumption is not the only one that could be made), and a matrix of probabilities that the patch encountered has a given state, and hence will have its state augmented through immigrating by one invading individual in a given state. We have also sketched how to include, e.g., a mechanism of active patch selection by migrants. However, such models with conditional dispersal quickly become too complex and parameter-rich, and thus had better be avoided in first explorations.

Similar to immigration, we have described emigration from the patches into the disperser pool through a matrix of expected rates at which patches in certain states release individuals of certain types into the disperser pool. While we have discussed the construction of this matrix with the help of a plausible decomposition, this could be done in a different and more complex way. In particular, one could choose to include per capita emigration rates that depend on a patch's state. In such cases, the possible effects on sojourn times in the patches can be considered analogously to how we considered the possible effects of conditional immigration on sojourn times in the disperser pool.

As in the case of 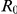 for well-mixed populations, there is an important distinction between deterministic and stochastic assessments of invasion success. When the number of individuals is large, chance effects resulting from fluctuations in their number or distribution will be averaged out. In contrast, if the population size is relatively small, one can only say that 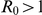 implies a positive probability that a major outbreak occurs [1]. In the case of metapopulations, analogous arguments apply with regard to the number of patches. This is why we have assumed a large number of patches, which implies deterministic dynamics at the metapopulation level, despite the fact that within each patch dynamics are stochastic.

Being able to determine whether an infectious agent can invade a metapopulation is useful for many purposes, the most important of which is the evaluation of eradication strategies. Despite the underlying simplifying assumptions, such as the large (infinite) number of patches and their equal connectivity, the invasion indicator 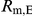 can give valuable insights into the invasion potential of an infectious agent. In this study, we have shown this with regard to the simplest, and hence most parameter-sparse, model for infectious-disease dynamics, given by an 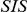-model. As the method we have developed here is much more general, as explained in the paragraphs above, it opens up the possibility to study many more realistic scenarios of infectious-disease invasion and persistence in a similar manner.

## Supporting Information

Appendix S1 (PDF)

Appendix S2 (PDF)
