# Supplementary material for: Invasion and Persistence of Infectious Agents in Fragmented Host Populations
Source: PLoS One. 2011 Sep 30;6(9):e24006. doi: 10.1371/journal.pone.0024006 (PMC3184079; doi:10.1371/journal.pone.0024006)
Supplement: Appendix S1 — (PDF) [file pone.0024006.s001.pdf]

Appendix S1:
Numerical procedure for finding the metapopulation equilibrium

Finding the equilibrium $(\hat{\mathbf{p}}, \hat{\mathbf{d}})$ requires solving Eqs. 13. We use the following scheme of iterative substitution, beginning with an initial guess $\mathbf{d}_0$ for the disperser-pool state:

0. Let $\mathbf{d} = \mathbf{d}_0$.
1. Construct $\mathbf{B}(\mathbf{d})$ and solve $\mathbf{B}(\mathbf{d})\mathbf{p} = 0$ for $\mathbf{p}$.
2. If $f(\mathbf{d}, \mathbf{p}) \neq 0$ up to numerical precision, solve $f(\mathbf{d}, \mathbf{p}) = 0$ for $\mathbf{d}$ and return to step 1;

   otherwise, let $\hat{\mathbf{p}} = \mathbf{p}$ and $\hat{\mathbf{d}} = \mathbf{d}$, and stop.

Advice for step 1: The equation in step 1 can be solved by using a procedure offered in many numerical software to find the eigenvector corresponding to the zero eigenvalue of $\mathbf{B}$ (i.e., its null space). Normalizing this eigenvector gives the estimate for $\mathbf{p}$. Alternatively, one can exploit the sparseness of $\mathbf{B}$ with the following procedure:

a) Find $b_{\max} = \max_j\{-b_{jj}\}$, where b_{jj} are the diagonal elements of $\mathbf{B}$.

b) Construct $\mathbf{C} = \mathbf{B} + b_{\max}\mathbf{I}$.

c) From an initial guess $\mathbf{q}_0$, iterate $\mathbf{q}_{n+1} = \mathbf{C}\mathbf{q}_n / \|\mathbf{q}_n\|$ to convergence; then let $\mathbf{p} = \mathbf{q}_{\text{final}} / \|\mathbf{q}_{\text{final}}\|$.

Advice for step 2: We find that for $\dim(\mathbf{d}) = m > 1$ the equation in step 2 is best solved by considering it in terms of the minimization problem $\mathbf{p} = \operatorname{argmin}_{\mathbf{d}'} \|f(\mathbf{d}', \mathbf{p})\|^2$ and using a multi-dimensional optimization procedure to obtain the estimate for $\mathbf{d}$. Since this scheme depends on multi-dimensional optimization, it may take some experimentation with initial values to obtain satisfactory convergence to a non-trivial equilibrium.
