# Supplementary material for: Invasion and Persistence of Infectious Agents in Fragmented Host Populations
Source: PLoS One. 2011 Sep 30;6(9):e24006. doi: 10.1371/journal.pone.0024006 (PMC3184079; doi:10.1371/journal.pone.0024006)
Supplement: Appendix S2 — (PDF) [file pone.0024006.s002.pdf]

Appendix S2:
Numerical procedure for obtaining the matrix product UT

For calculating $\mathbf{R} = \mathbf{VUTS}$ in Eq. 3 using $\mathbf{T} = -\tilde{\mathbf{B}}^{-1}$ in Eq. 10, the product $-\mathbf{U}\tilde{\mathbf{B}}^{-1}$ is needed. Here we provide a simple and efficient procedure for obtaining this matrix product. This procedure exploits the sparseness of $\tilde{\mathbf{B}}$ and uses a decomposition of $\tilde{\mathbf{B}}$ into a diagonal matrix and a matrix in which all elements are smaller than 1. Consequently, the inversion of the former matrix is trivial, while the inverse of the latter matrix can be computed as a geometric series (see the discussion of the structure of $\mathbf{B}$ following Eqs. 2):

1. Construct a diagonal matrix $\mathbf{D}$ containing the negative diagonal of $\tilde{\mathbf{B}}$, with diagonal elements $d_{jj} = -\tilde{b}_{jj}$ (which are all positive, since the diagonal elements of $\tilde{\mathbf{B}}$ are negative).

2. Construct $\mathbf{H} = \mathbf{D}^{-1}(\tilde{\mathbf{B}}+\mathbf{D})$, with diagonal elements $h_{jj} = 0$ and off-diagonal elements $h_{ij} = \tilde{b}_{ij}/\left|\tilde{b}_{jj}\right|$ (hence $0 \leq h_{jj} < 1$), so that $\tilde{\mathbf{B}} = -\mathbf{D}(\mathbf{I} - \mathbf{H})$.

3. Construct $\mathbf{D}^{-1}$ as the diagonal matrix with diagonal elements $1/d_{jj} = -1/\tilde{b}_{jj}$.

4. Since $(\mathbf{I} - \mathbf{H})^{-1}$ is the limit of a geometric series, $(\mathbf{I} - \mathbf{H})^{-1} = \sum_{n=0}^{\infty} \mathbf{H}^n$, $\mathbf{U}(\mathbf{I} - \mathbf{H})^{-1}$ is directly computed by iterating $\mathbf{Z}_n = \mathbf{U}+\mathbf{Z}_{n-1}\mathbf{H}$, with $\mathbf{Z}_0 = \mathbf{U}$, to convergence (which is guaranteed, because $\mathbf{Z}_n - \mathbf{Z}_{n-1} = \mathbf{Z}_0\mathbf{H}^n$, so $\mathbf{Z}_n$ is a Cauchy sequence).

5. Calculate $-\mathbf{U}\tilde{\mathbf{B}}^{-1} = \mathbf{U}(\mathbf{I} - \mathbf{H})^{-1}\mathbf{D}^{-1}$.
